# A data-driven priority assessment and deployment framework for medical equipment maintenance in a tertiary hospital

**DOI:** 10.3389/frai.2026.1791935

**Published:** 2026-04-14

**Authors:** Chenjian Ye, Sunzhong Lin, Li Yanjun, Pengcheng Zhou

**Affiliations:** 1 Second Affiliated Hospital and Yuying Children's Hospital of Wenzhou Medical University, Wenzhou, China; 2 Wenzhou People's Hospital, Wenzhou, China

**Keywords:** corrective maintenance, hospital operations management, machine learning, maintenance prioritization, medical equipment maintenance, preventive maintenance

## Abstract

**Background:**

Effective maintenance management of medical equipment is essential to ensure patient safety, operational continuity, and cost control in hospitals. Traditional experience-based maintenance strategies often fail to capture the dynamic risk profiles of heterogeneous equipment, particularly in large healthcare institutions. Data-driven approaches may improve maintenance prioritization, yet evidence from real-world hospital deployment remains limited.

**Methods:**

We developed and implemented a machine learning–assisted priority evaluation system for medical equipment maintenance in a tertiary hospital. Separate priority assessment frameworks were established for preventive maintenance (PM) and corrective maintenance (CM), each incorporating domain-specific features and weighted scoring schemes. Multiple machine learning models, including logistic regression, decision tree, support vector machine, naïve Bayes, and XGBoost, were trained and evaluated using a stratified training–testing split. Model performance was assessed using accuracy, precision, recall, F1-score, receiver operating characteristic (ROC) curves, and confusion matrices. The optimal model was deployed into the hospital maintenance workflow and evaluated in a parallel controlled implementation.

**Results:**

A total of 9,924 medical devices were included, comprising 8,967 devices with preventive maintenance (PM) records and 957 devices with corrective maintenance (CM) records. Devices were stratified into low-, medium-, and high-urgency groups using clustering-derived labels. Among the five machine learning algorithms evaluated, XGBoost achieved the best performance, with a testing accuracy of 0.9379 in the PM dataset and 0.8646 in the CM dataset. In the real-world deployment phase (2025.1.2–2025.12.25), 830 devices in the intervention campus and 849 devices in the control campus were compared. The intervention campus showed lower proportions of failures, recurrence, and unplanned maintenance events, and a lower overall maintenance cost ratio than the control campus (4.8% vs. 7.3%).

**Conclusion:**

This study demonstrates the feasibility and practical value of deploying a machine learning–assisted priority evaluation system for medical equipment maintenance in a real hospital environment. By distinguishing preventive and corrective maintenance scenarios and integrating model outputs into routine workflows, the proposed framework supports more efficient, consistent, and cost-effective maintenance decision-making.

## 1 Introduction

Medical equipment is fundamental to modern healthcare delivery, underpinning diagnostic accuracy, therapeutic effectiveness, and patient safety (Aronson et al., 2020). With the rapid expansion of healthcare systems and the increasing complexity of medical technologies, hospitals are required to manage large inventories of heterogeneous equipment while ensuring high reliability, availability, and cost-effectiveness (Webber et al., 2020). Inefficient maintenance strategies may lead to frequent equipment failures, prolonged downtime, increased operational costs, and disruption of clinical services (Perry and Malkin, 2011).

In routine hospital practice, medical equipment maintenance management is still largely driven by fixed schedules, manufacturer recommendations, or the experience of clinical engineers (Wang et al., 2006). Although such approaches are easy to implement, they often fail to capture the dynamic risk profiles of individual devices, including equipment age, functional criticality, historical failure patterns, maintenance complexity, and operational context (Iadanza et al., 2019). As a result, low-risk equipment may be over-maintained, while high-risk equipment may not receive timely intervention, particularly in large tertiary hospitals managing thousands of devices simultaneously (Sezdi, 2016; Hamdi et al., 2012).

Recent advances in data analytics and machine learning have provided new opportunities to improve maintenance prioritization through data-driven decision-making (Li et al., 2025). Prior studies have explored the use of machine learning techniques to assess equipment criticality and predict maintenance needs. Notably, Zamzam et al. (2021) proposed a comprehensive prioritisation and predictive framework for medical equipment maintenance, integrating clustering and supervised learning methods to support preventive maintenance, corrective maintenance, and replacement decisions. Their work demonstrated the potential of data-driven prioritisation to enhance consistency and reduce reliance on subjective judgement in equipment management.

Despite these advances, several challenges remain in translating prioritisation models into real-world hospital operations (Rongrong, 2022). First, many studies primarily focus on model development and performance metrics, with limited emphasis on clinical deployment and workflow integration. Second, preventive maintenance (PM) and corrective maintenance (CM) are often addressed within a unified framework, despite their distinct decision logic, urgency characteristics, and resource allocation requirements in daily practice. Third, the downstream impact of priority-based maintenance systems on execution compliance, equipment reliability, and economic outcomes has not been sufficiently evaluated in real hospital environments (Abd Wahab et al., 2024).

To address these gaps, the present study develops and implements a machine learning–assisted priority evaluation system for medical equipment maintenance in a tertiary hospital setting. Building upon data-driven prioritisation concepts, we designed separate yet methodologically consistent scoring and weighting schemes for preventive and corrective maintenance, reflecting their different operational objectives. The system was fully deployed within the hospital maintenance workflow to support automated priority classification and maintenance decision-making.

Using large-scale hospital equipment data, we systematically evaluated the proposed system from multiple perspectives, including priority distribution, maintenance execution, reliability outcomes, and economic performance. By integrating machine learning–based prioritisation with real-world deployment and outcome assessment, this study aims to provide practical evidence for the feasibility and value of intelligent maintenance management systems in hospital settings.

## 2 Methods

### 2.1 Study design and data source

This study adopted a three-phase methodological framework, consisting of:

1. Retrospective extraction of medical equipment baseline characteristics and maintenance data;
2. Unsupervised clustering and supervised machine learning classification to construct a maintenance urgency prediction model; and
3. Real-world quasi-experimental validation in two hospital campuses.

The study was conducted at the Second Affiliated Hospital of Wenzhou Medical University, a large tertiary grade A general hospital in Wenzhou, China. The hospital had approximately 3,200 beds and a relatively mature digital maintenance environment supporting structured capture of equipment operation and maintenance data. The model was trained using data collected between January 1, 2024, and December 31, 2024, including equipment age records, and was applied during the period from January 2, 2025, to December 25, 2025. Devices included were fixed hospital-owned assets with complete maintenance history. Exclusion criteria involved *in vitro* diagnostic instruments, low-value consumables, dormant devices without maintenance activity, and devices missing core maintenance attributes.

A total of 9,924 devices were eligible after screening. Depending on maintenance patterns, devices were categorized into two analytical pathways:

- A PM dataset consisting of 8,967 devices subjected to scheduled maintenance tracking; and
- A CM dataset consisting of 957 devices with complete failure repair records.

**Figure 1:**
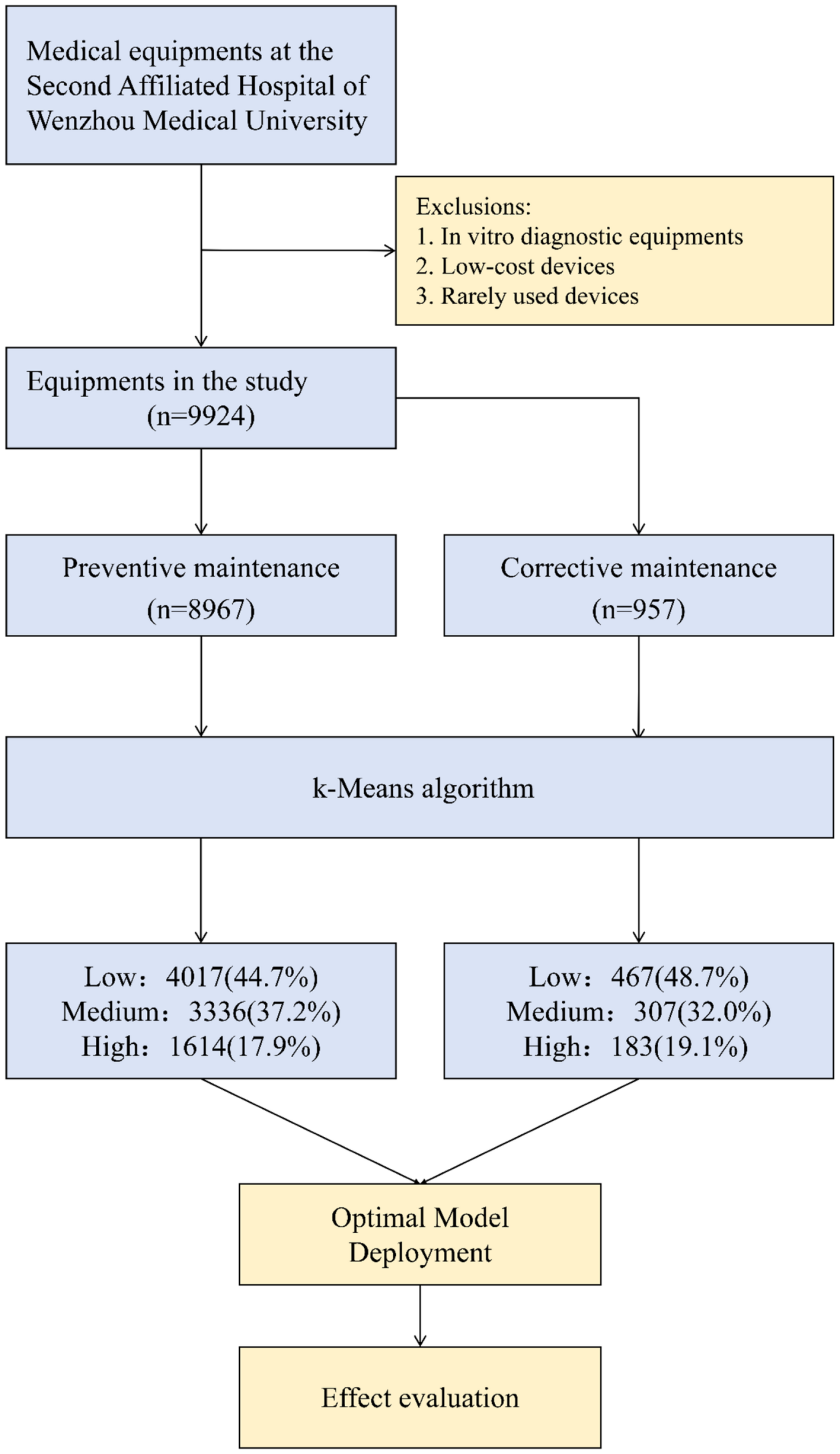
Flowchart.

Both datasets were modeled independently to capture their distinct operational patterns, while maintaining consistent variable definitions and coding rules. An overview of the device selection process, analytical workflow, and model deployment pipeline is illustrated in Figure 1.

### 2.2 Preventive and corrective maintenance operational definition

PM was defined as planned, periodic interventions aimed at sustaining device performance and preventing failure. PM records included scheduled intervals, completion status, maintenance complexity, and the number of missed preventive maintenance tasks. PM performance was further characterized using downtime and historical failure patterns.

CM was defined as unplanned servicing triggered by device malfunction or performance interruption. CM records included full repair workflows: event reporting, response initiation, troubleshooting duration, material consumption, and completion confirmation. From these timestamps, maintenance efficiency metrics such as response time, repair time, and unplanned downtime were calculated. Additionally, failure count and maintenance cost ratio (maintenance cost/asset cost) were used to capture operational burdens and lifecycle impact.

Across both pathways, operational features were standardized to numerically encode device functional classifications (monitoring, diagnostic, treatment, life-support, or others), device age, backup unit availability, fault patterns, and resource utilization. Integration of PM and CM operational perspectives enabled comprehensive characterization of device reliability and real-world maintenance demands.

### 2.3 Feature engineering and variable encoding

A unified feature set was developed based on the original fields extracted from the hospital equipment management system, covering device attributes, maintenance categories, maintenance complexity, and multiple continuous operational indicators. Detailed definitions and coding rules are summarized in Table 1. All features were numerically encoded and standardized prior to modeling to ensure comparability across variables with different units and scales.

**Table 1:** Definition and encoding criteria of features used for model development.

Features	Criteria
Equipment age	The unit is years, continuous features
Function	Miscellaneous (0) Monitor (1) Diagnostic (2) Therapeutic (3) Life support (4)
Backup or alternative unit	No (0) Yes (1)
Maintenance requirement	Routine Inspection (0) PM (Once annually) (1) PM (Once annually) and Calibration (2) PM (Twice annually) and Calibration (Twice annually) (3)
Maintenance complexity	Self-inspection (0) Monthly inspection (1) Daily inspection (2)
Response time	The unit is days, continuous features
Repair time	The unit is days, continuous features
Downtime	The unit is days, continuous features
Number of failures	The unit is ‘times’, continuous features
Maintenance cost	The unit is percentage, continuous features
Number of missed Planned Preventive Maintenance (PM)	The unit is ‘times’, continuous features

### 2.4 Clustering analysis and urgency label construction

Unsupervised clustering was performed separately on the PM and CM datasets to construct reference labels for maintenance urgency. All variables were numerically encoded and standardized according to the definitions in Table 1 prior to analysis. K-means clustering was then applied in the unified feature space composed of device characteristics and maintenance-related operational variables.

To determine the optimal number of clusters, candidate solutions with K = 2, 3, 4, and 5 were evaluated in both datasets using the Silhouette score and Davies–Bouldin index. The final choice of K = 3 was based on the combined consideration of quantitative clustering performance and practical interpretability for maintenance prioritization. For the selected K = 3 solution, the Silhouette scores were 0.70 in the PM dataset and 0.67 in the CM dataset, while the Davies–Bouldin indices were 0.48 and 0.50, respectively, indicating acceptable cluster compactness and separation. Low-dimensional PCA projections were additionally generated to visualize the separation of urgency groups.

The resulting clusters were treated as pseudo–ground truth urgency labels for subsequent supervised learning. We acknowledge that these labels represent data-driven statistical structure rather than an external clinical gold standard. Therefore, the downstream classification models should be interpreted as predicting cluster-defined urgency categories rather than directly reproducing independently validated real-world urgency.

To support downstream interpretation, a feature-weighted scoring system was constructed separately for PM and CM (Supplementary Tables S1–S4). Weight assignment was based on a structured expert-informed procedure integrating Delphi consultation with risk-contribution assessment. Five senior clinical engineering experts, each with more than 10 years of experience in medical equipment maintenance management in tertiary hospitals, independently evaluated the relative importance of candidate variables on a 1–10 scale. The scoring framework considered three common dimensions: potential clinical impact, likelihood of failure or service disruption, and expected maintenance resource demand. Inter-expert agreement was assessed using Kendall’s coefficient of concordance, which was 0.87 for the PM pathway and 0.89 for the CM pathway, indicating good consistency. Variables with larger scoring discrepancies were discussed and harmonized through consensus review. Original variables were then converted into ordinal sub-scores and aggregated using pathway-specific feature weights. These weighted scores were not involved in the clustering procedure and were used solely for descriptive analysis and interpretability support in the Results section.

To evaluate the robustness of the weighting scheme, we performed a weight-perturbation sensitivity analysis. For each pathway, feature weights were independently increased or decreased by 10, 20, and 30%, while preserving the original expert-assigned range using lower and upper bounds of 1 and 10. Under each perturbation scenario, total weighted scores were recalculated and urgency classifications were re-derived. Agreement with the original classification was quantified using the classification consistency rate. In addition, the association between the weighted total score and the cluster-derived urgency label was assessed using Spearman’s rank correlation coefficient.

### 2.5 Prediction model development

Supervised learning models were developed to predict maintenance urgency levels (low, medium, high) using the pseudo–ground truth labels derived from clustering. All standardized features were used as predictors. Each dataset was randomly divided into a training set (70%) and a testing set (30%).

Five machine learning algorithms—logistic regression, decision tree, support vector machine (SVM), naïve Bayes, and XGBoost—were trained for comparison. Hyperparameters were optimized using grid search combined with five-fold cross-validation to reduce overfitting. Predictive performance was assessed using accuracy, class-wise precision, recall, and F1-score, as well as macro-F1, weighted-F1, confusion matrices, and one-vs-rest ROC curves with their corresponding AUC values. XGBoost was ultimately selected as the final model for deployment based on its superior overall performance.

### 2.6 Model deployment and workflow integration

Prior to real-world implementation, a structured deployment framework was established to integrate the prediction models into routine maintenance workflows. During the 12-month (2025.1.2–2025.12.25) intervention period, both PM and CM urgency models were fully deployed in the intervention campus, while the control campus continued conventional manual scheduling.

For PM, all equipment in the intervention campus was automatically classified into low-, medium-, or high-urgency categories based on model outputs, with corresponding recommendations on maintenance frequency and completion timeliness. Engineers recorded whether model recommendations were followed and whether maintenance tasks were completed within the recommended time window.

For CM, urgency levels were generated at the time of failure based on CM-specific weighted features. Engineers documented whether model-guided actions were adopted, as well as actual response time, repair duration, and resource utilization.

### 2.7 Outcome measures and statistical analysis

To evaluate the impact of model deployment, prespecified reliability outcomes included failure rate, recurrence, unplanned maintenance events, and planned maintenance completion rate. Economic outcomes included overall maintenance cost ratio and category-specific cost ratios across monitoring, diagnostic, therapeutic, and life-support equipment.

For post-deployment comparisons between the intervention and control campuses, categorical outcomes were compared using chi-square tests or Fisher’s exact tests as appropriate, and continuous outcomes were compared using independent-samples t-tests. Effect sizes with 95% confidence intervals were additionally calculated for the principal deployment outcomes. A two-sided *p* value < 0.05 was considered statistically significant. All analyses were performed using Python 3.10.16, primarily with the following libraries: pandas, numpy, scikit-learn, matplotlib, xgboost, seaborn, plotly, statsmodels, and scipy.

## 3 Results

### 3.1 Device inventory overview and urgency-stratified characteristics

A total of 9,924 medical devices from the Second Affiliated Hospital of Wenzhou Medical University were included in the analysis, covering a wide range of functional categories and distributed across four hospital campuses. PM records were available for 8,967 devices, while 957 devices had complete CM records. Detailed device counts and functional distributions by campus are provided in Supplementary Tables S5, S6.

Based on clustering-derived labels, devices in the PM and CM datasets were separately stratified into low-, medium-, and high-urgency groups. Cluster validation analyses supported the selected three-group urgency structure in both datasets. For the final K = 3 solution, the Silhouette scores were 0.70 for PM and 0.67 for CM, while the Davies–Bouldin indices were 0.48 and 0.50, respectively. Comparative analyses across candidate solutions (K = 2, 3, 4, and 5) showed that K = 3 provided the most favorable overall balance between cluster compactness, inter-cluster separation, and operational interpretability. PCA-based visualizations further demonstrated acceptable separation among the three urgency groups, although partial overlap remained in some boundary cases, as expected for real-world maintenance data (Supplementary Table S7 and Supplementary Figures S1, S2). In the PM dataset, low-urgency devices accounted for approximately 45%, medium-urgency for 37%, and high-urgency for 18% of devices. In the CM dataset, the corresponding proportions were approximately 49, 32, and 19%, respectively. The distributions of key operational and maintenance-related characteristics across urgency levels are summarized in Table 2 (PM) and Table 3 (CM).

**Table 2:** Baseline characteristics of preventive maintenance devices across urgency levels identified by clustering.

Variables	Low urgency	Medium urgency	High urgency
Downtime			
0	2,772 (69.01%)	1997 (59.86%)	481 (29.8%)
[1, 7]	710 (17.67%)	847 (25.39%)	839 (51.98%)
>7	535 (13.32%)	492 (14.75%)	294 (18.22%)
Function			
Miscellaneous	2,908 (72.39%)	954 (28.6%)	518 (32.09%)
Monitor	151 (3.76%)	1777 (53.27%)	123 (7.62%)
Diagnostic	37 (0.92%)	208 (6.24%)	90 (5.58%)
Therapeutic	795 (19.79%)	287 (8.6%)	641 (39.71%)
Life support	126 (3.14%)	110 (3.3%)	242 (14.99%)
Backup or alternative unit			
No	1,386 (34.5%)	1,139 (34.14%)	1,254 (77.7%)
Yes	2,631 (65.5%)	2,197 (65.86%)	360 (22.3%)
Equipment age			
≤5	1974 (49.14%)	1709 (51.23%)	624 (38.66%)
[5, 10]	1,409 (35.08%)	1,023 (30.67%)	656 (40.64%)
>10	634 (15.78%)	604 (18.11%)	334 (20.69%)
Number of failures			
0	2,850 (70.95%)	2,175 (65.2%)	492 (30.48%)
[1, 5]	953 (23.72%)	868 (26.02%)	959 (59.42%)
> 5	214 (5.33%)	293 (8.78%)	163 (10.1%)
Maintenance complexity			
Self-inspection	414 (10.31%)	237 (7.1%)	25 (1.55%)
Monthly inspection	3,570 (88.87%)	1,312 (39.33%)	426 (26.39%)
Daily inspection	33 (0.82%)	1787 (53.57%)	1,163 (72.06%)
Maintenance requirement			
0	1993 (49.61%)	115 (3.45%)	241 (14.93%)
1	1986 (49.44%)	933 (27.97%)	1,112 (68.9%)
2	18 (0.45%)	533 (15.98%)	250 (15.49%)
3	20 (0.5%)	1755 (52.61%)	11 (0.68%)
PM			
0	3,034 (75.53%)	1871 (56.09%)	540 (33.46%)
[1, 3]	892 (22.21%)	1,392 (41.73%)	1,008 (62.45%)
>3	91 (2.27%)	73 (2.19%)	66 (4.09%)

Percentages are column-based and were calculated within each urgency group; therefore, percentages across a given row do not necessarily sum to 100%. Minor deviations from 100% are due to rounding.

**Table 3:** Baseline characteristics of corrective maintenance devices across urgency levels identified by clustering.

Variables	Low urgency	Medium urgency	High urgency
Equipment age			
<5	195 (41.76%)	87 (28.34%)	48 (26.23%)
[5, 10]	219 (46.9%)	142 (46.25%)	65 (35.52%)
>10	53 (11.35%)	78 (25.41%)	70 (38.25%)
Function			
Miscellaneous	340 (72.81%)	91 (29.64%)	31 (16.94%)
Monitor	23 (4.93%)	155 (50.49%)	27 (14.75%)
Diagnostic	6 (1.28%)	10 (3.26%)	6 (3.28%)
Therapeutic	87 (18.63%)	30 (9.77%)	69 (37.7%)
Life support	11 (2.36%)	21 (6.84%)	50 (27.32%)
Backup or alternative unit			
No	71 (15.2%)	118 (38.44%)	126 (68.85%)
Yes	396 (84.8%)	189 (61.56%)	57 (31.15%)
Number of failures			
1	206 (44.11%)	74 (24.1%)	38 (20.77%)
[2, 5]	223 (47.75%)	146 (47.56%)	84 (45.9%)
>5	38 (8.14%)	87 (28.34%)	61 (33.33%)
Maintenance complexity			
Self-inspection	42 (8.99%)	11 (3.58%)	5 (2.73%)
Monthly inspection	368 (78.8%)	115 (37.46%)	52 (28.42%)
Daily inspection	57 (12.21%)	181 (58.96%)	126 (68.85%)
Response time			
1	285 (61.03%)	173 (56.35%)	95 (51.91%)
[2, 7]	82 (17.56%)	69 (22.48%)	33 (18.03%)
>7	100 (21.41%)	65 (21.17%)	55 (30.05%)
Repair time			
1	235 (50.32%)	181 (58.96%)	108 (59.02%)
[2, 7]	127 (27.19%)	57 (18.57%)	30 (16.39%)
>7	105 (22.48%)	69 (22.48%)	45 (24.59%)
Maintenance cost (%)			
≤5	403 (86.3%)	256 (83.39%)	149 (81.42%)
[5, 10]	51 (10.92%)	35 (11.4%)	7 (3.83%)
>10	13 (2.78%)	16 (5.21%)	27 (14.75%)

Percentages are column-based and were calculated within each urgency group; therefore, percentages across a given row do not necessarily sum to 100%. Minor deviations from 100% are due to rounding.

**Figure 2:**
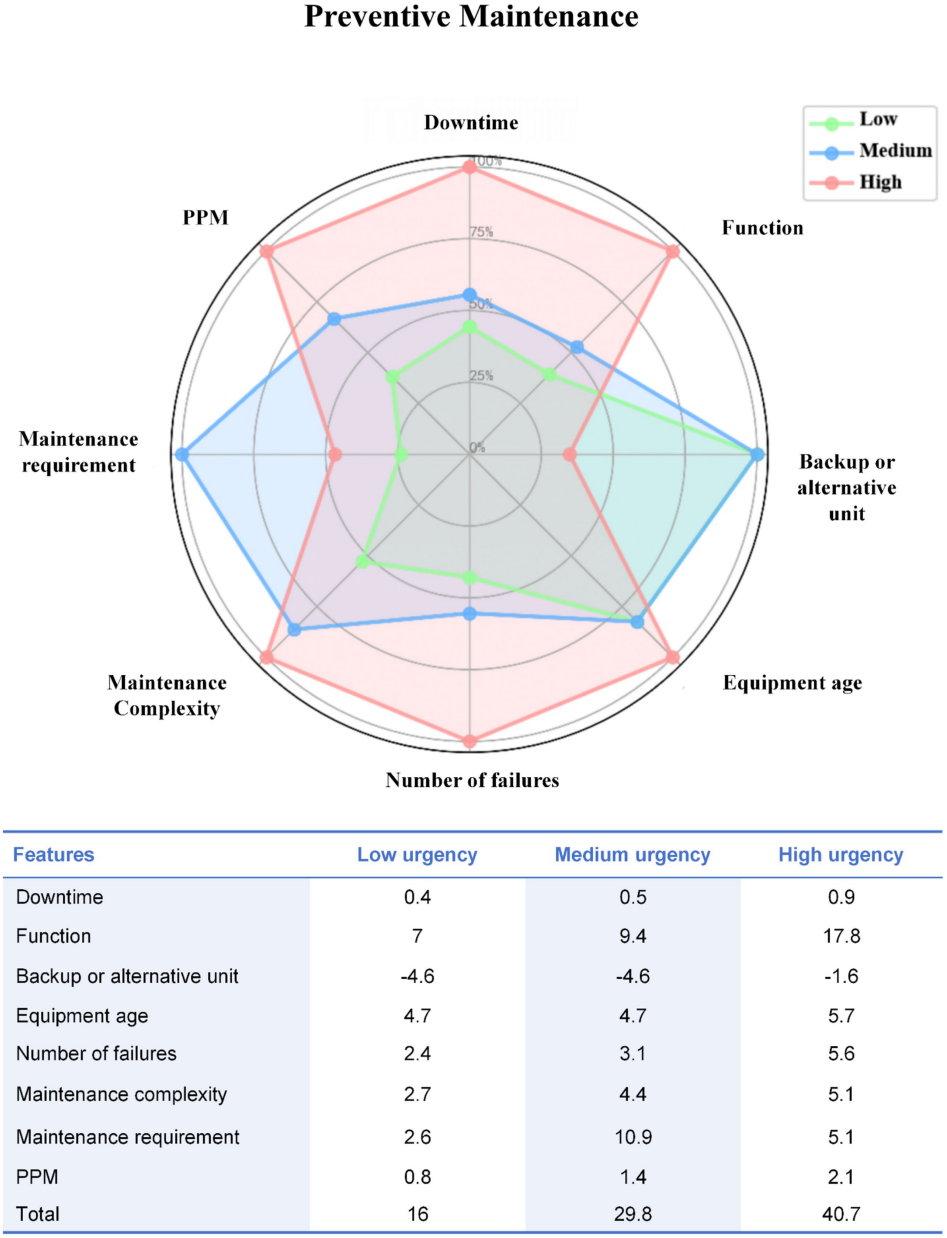
Radar chart of weighted feature scores across urgency levels in preventive maintenance.

Within the PM dataset, a clear gradient of operational burden and maintenance demand was observed across urgency categories. High-urgency devices more frequently exhibited advanced service age, greater maintenance complexity, and higher failure burden compared with low-urgency devices, while medium-urgency devices consistently showed intermediate profiles. Feature-weighted score distributions further supported this stratification, with total scores increasing monotonically from low to high urgency (Figure 2).

**Figure 3:**
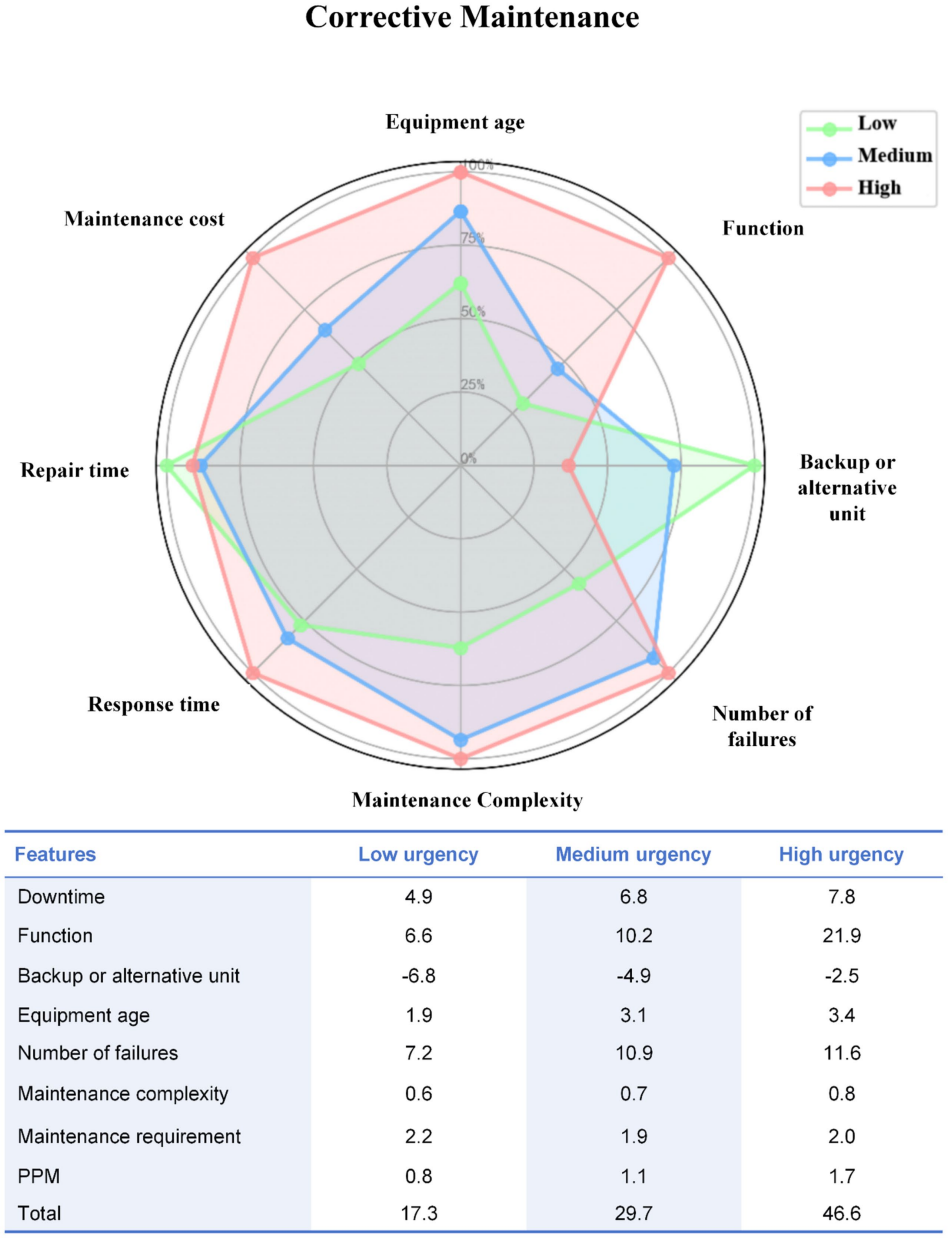
Radar chart of weighted feature scores across urgency levels in corrective maintenance.

Similar patterns were observed in the CM dataset. Low-urgency devices were more likely to be newer and to have backup units available, whereas high-urgency devices were characterized by limited redundancy, higher failure frequency, longer repair duration, and increased maintenance cost burden. Feature-weighted scores demonstrated clear separation across urgency levels, reflecting multidimensional escalation of maintenance risk (Figure 3).

The interpretability of the weighted scoring system was further examined quantitatively. Weighted total scores showed a strong positive correlation with cluster-derived urgency labels in both pathways, with Spearman’s *ρ* = 0.89 (*p* < 0.001) for PM and *ρ* = 0.91 (*p* < 0.001) for CM, indicating high concordance between score-based stratification and unsupervised urgency grouping. Sensitivity analyses further demonstrated the robustness of the weighting scheme. Across ±10 and ±20% perturbation scenarios, classification consistency remained above 92% in both datasets, and even under ±30% perturbation, consistency remained above 86%. Importantly, no direct cross-level misclassification between low- and high-urgency groups was observed in any perturbation scenario; discrepancies were limited to a small proportion of adjacent-category overlap (Supplementary Tables S8–S10).

Collectively, these results indicate that urgency stratification derived from unsupervised clustering captures meaningful gradients in device condition, maintenance complexity, and operational risk across both preventive and corrective maintenance scenarios.

### 3.2 Machine learning model performance

**Figure 4:**
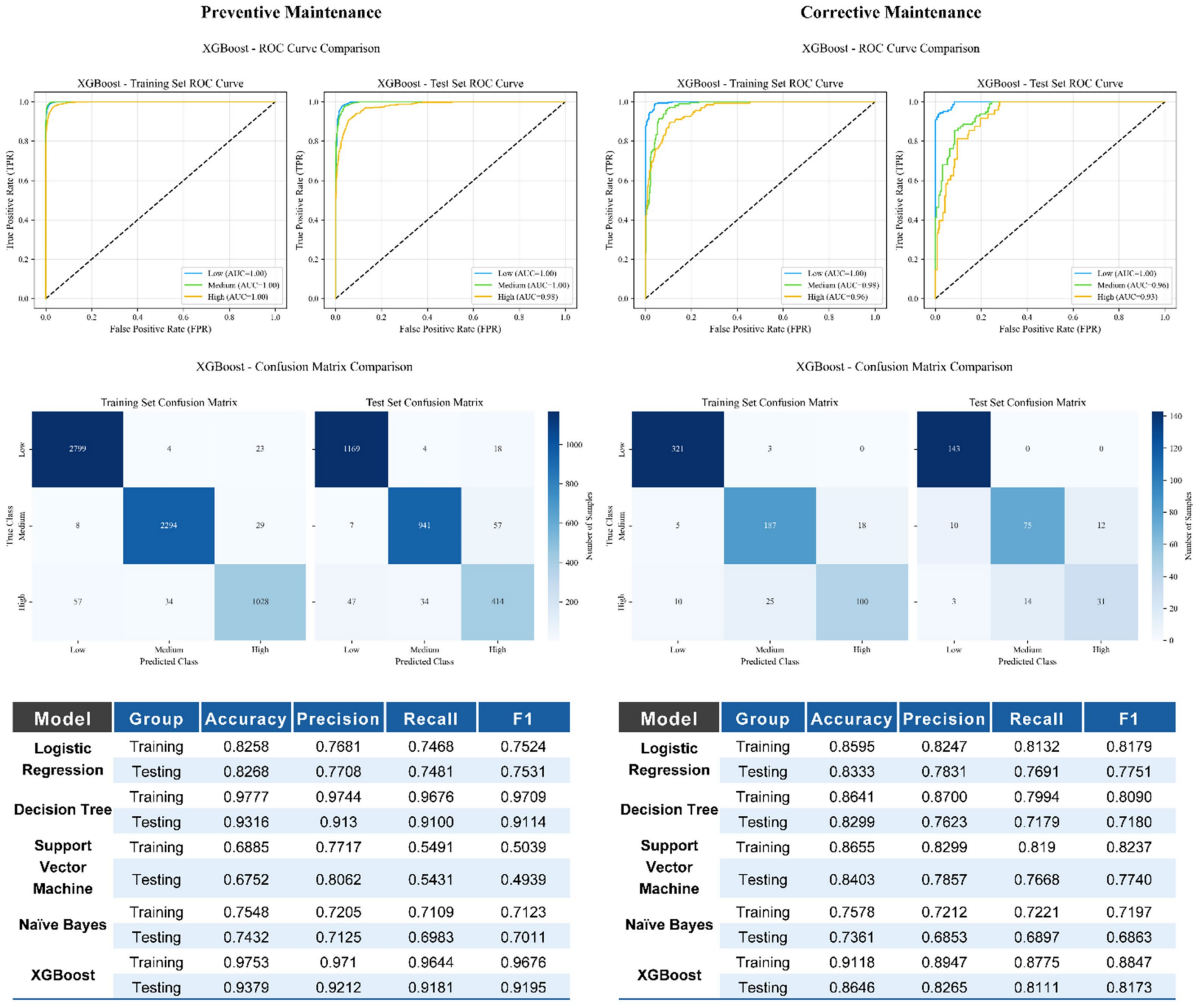
Performance of the XGBoost and other model for urgency classification in preventive and corrective maintenance.

Five machine learning algorithms were trained and evaluated on both the PM and CM datasets using a 70/30 train–test split. A detailed comparison of model performance metrics, including accuracy, precision, recall, and F1-score, is provided in Figure 4, with additional results presented in the Supplementary Figures S3, S4.

Across both maintenance scenarios, XGBoost consistently demonstrated the best overall classification performance and was therefore selected as the final model for deployment. In the PM dataset, XGBoost achieved a testing accuracy of 0.9379, with precision, recall, and F1-score all exceeding 0.91, indicating strong and balanced discrimination across urgency levels. In the CM dataset, XGBoost likewise outperformed the other models, reaching a testing accuracy of 0.8646, with all major performance metrics remaining above 0.81.

Compared with XGBoost, the remaining models showed inferior or less stable performance. Logistic regression and decision tree models provided moderate discrimination, while naïve Bayes and support vector machine exhibited higher misclassification rates, particularly between medium- and high-urgency categories (Supplementary Figures S3, S4).

Across both maintenance scenarios, XGBoost consistently demonstrated the best overall performance and was therefore selected as the final model for deployment. In the PM dataset, the XGBoost model achieved a testing accuracy of 0.9379, with a macro-F1 of 0.884 and a weighted-F1 of 0.918. In the CM dataset, the corresponding testing accuracy was 0.8646, with a macro-F1 of 0.805 and a weighted-F1 of 0.842. Class-wise evaluation further showed that discrimination was strongest for low-urgency devices and decreased gradually for medium- and high-urgency categories, particularly in the CM setting. Detailed class-wise precision, recall, and F1-scores are provided in Supplementary Table S11 and Supplementary Figures S5, S6.

Overall, these results demonstrate that XGBoost provides the most accurate and stable urgency classification across both preventive and corrective maintenance tasks, justifying its selection as the final prediction model for real-world deployment.

### 3.3 Model deployment and real-world evaluation

During 2025.1.2–2025.12.25, a total of 830 devices from the Longwan campus (intervention group) and 849 devices from the Lucheng campus (control group) were included in the real-world comparison. As shown in Table 4, there were no statistically significant baseline differences between the two campuses in terms of equipment age, functional category, downtime, availability of backup units, number of failures, maintenance complexity, or missed PM events (all *p* > 0.05), indicating good comparability before model deployment.

**Table 4:** Baseline characteristics of devices in the intervention and control campuses.

Characteristic	Longwan campus (*N* = 830)	Lucheng campus (*N* = 849)	*P*
Equipment age (years)			0.478
≤ 5	410 (49.4%)	395 (46.53%)	
[5, 10]	320 (38.55%)	342 (40.28%)	
> 10	100 (12.05%)	112 (13.19%)	
Function			0.516
Monitoring	585 (70.48%)	596 (70.2%)	
Diagnostic	66 (7.95%)	77 (9.07%)	
Therapeutic	35 (4.22%)	44 (5.18%)	
Life support	144 (17.35%)	132 (15.55%)	
Subcategory			0.534
Ventilator	91 (10.96%)	93 (10.95%)	
Infant incubator	22 (2.65%)	18 (2.12%)	
Patient monitor	585 (70.48%)	596 (70.2%)	
Hemodialysis	35 (4.22%)	44 (5.18%)	
Ultrasound	66 (7.95%)	77 (9.07%)	
Anesthesia machine	31 (3.73%)	21 (2.47%)	
Downtime (days)			0.403
0	416 (50.12%)	418 (49.23%)	
[1, 7]	245 (29.52%)	236 (27.8%)	
> 7	169 (20.36%)	195 (22.97%)	
Backup or alternative unit			0.957
No	170 (20.48%)	172 (20.26%)	
Yes	660 (79.52%)	677 (79.74%)	
Number of failures			0.988
0	434 (52.29%)	442 (52.06%)	
1	177 (21.33%)	179 (21.08%)	
[2, 5]	121 (14.58%)	123 (14.49%)	
> 5	98 (11.81%)	105 (12.37%)	
Maintenance complexity			0.960
Self-inspection	75 (9.04%)	79 (9.31%)	
Monthly inspection	398 (47.95%)	410 (48.29%)	
Daily inspection	357 (43.01%)	360 (42.4%)	
Maintenance requirement			0.484
Routine inspection	48 (5.78%)	30 (3.53%)	
PM once annually	182 (21.93%)	182 (21.44%)	
PM once annually + calibration	38 (4.58%)	61 (7.18%)	
PM twice annually + calibration	562 (67.71%)	576 (67.84%)	
Number of missed PM events			0.134
0	489 (58.92%)	466 (54.89%)	
[1, 3]	326 (39.28%)	359 (42.29%)	
> 3	15 (1.81%)	24 (2.83%)	

Model adoption in routine practice was high (Table 5). For preventive maintenance, the acceptance rate of model recommendations by engineers exceeded 98% across low-, medium-, and high-urgency devices, and the planned maintenance completion rate reached 98–100% within the study period. For corrective maintenance, all devices with failures in the intervention campus were managed according to the CM model recommendations, with a 100% adoption rate across all urgency levels.

**Table 5:** Adoption of model recommendations and planned maintenance completion rates.

Maintenance type	Urgency level	Total number	Adopted cases	Adoption rate	Planned completion rate
Preventive maintenance (PM)	Low urgency	62	61	99%	98%
	Medium urgency	636	623	98%	99%
	High urgency	132	132	100%	100%
Corrective maintenance (CM)	Low urgency	10	10	100%	–
	Medium urgency	50	50	100%	–
	High urgency	8	8	100%	–

With respect to reliability outcomes, the intervention campus showed lower proportions of devices with failures and failure recurrences compared with the control campus, and the share of unplanned maintenance events was also reduced (Table 6). Although the absolute differences were modest, the trends were consistently favorable across monitoring, diagnostic, therapeutic, and life-support equipment.

**Table 6:** Comparison of reliability and economic outcomes between the intervention and control campuses after model deployment.

Indicator	Intervention campus (*n* = 830)	Control campus (*n* = 849)	Effect size (95%CI)
Reliability outcomes			
Devices with failures, *n* (%)	68 (8.2%)	76 (8.9%)	RD = −0.7% (−1.57% ~ −0.03%)
Monitoring equipment failures, *n* (%)	46 (7.9%)	47 (7.9%)	RD = 0% (−1.24% ~ 1.25%)
Diagnostic equipment failures, *n* (%)	5 (7.5%)	2 (2.6%)	RD = 4.9% (0.52% ~ 9.28%)
Therapeutic equipment failures, *n* (%)	5 (14.2%)	12 (27.0%)	RD = -12.8% (−23.17% ~ −2.43%)
Life-support equipment failures, *n* (%)	12 (8.3%)	15 (11.3%)	RD = −3.0% (−5.88% ~ −0.11%)
Failure recurrence rate, *n* (%)	8 (11.5%)	15 (19.4%)	RD = −7.9% (−13.62% ~ −2.18%)
Proportion of unplanned maintenance (%)	4.2%	8.7%	RD = −4.5% (−5.21% ~ −3.79%)
Economic outcomes			
Maintenance cost ratio (%) – overall mean	4.8%	7.3%	MD = −2.5% (−2.86% ~ −2.12%)
Maintenance cost ratio (%) – monitoring equipment (mean)	3.2%	5.1%	MD = −1.9% (−2.24% ~ −1.56%)
Maintenance cost ratio (%) – diagnostic equipment (mean)	6.5%	9.8%	MD = −3.3% (−3.75% ~ −2.84%)
Maintenance cost ratio (%) – therapeutic equipment (mean)	7.1%	11.4%	MD = −4.3% (−4.89% ~ −3.71%)
Maintenance cost ratio (%) – life-support equipment (mean)	5.3%	8.2%	MD = −2.9% (−3.32% ~ −2.49%)

From an economic perspective, the maintenance cost ratio was markedly lower in the intervention group. The overall maintenance cost accounted for 4.8% of total equipment value in the intervention campus, compared with 7.3% in the control campus. When stratified by functional category, the intervention group also demonstrated lower average cost ratios for monitoring devices (3.2% vs. 5.1%), diagnostic equipment (6.5% vs. 9.8%), therapeutic devices (7.1% vs. 11.4%), and life-support equipment (5.3% vs. 8.2%). These findings suggest that integrating the urgency prediction model into maintenance workflows can improve maintenance efficiency while reducing economic burden.

## 4 Discussion

The present study developed and implemented a machine learning–assisted priority evaluation system for medical equipment maintenance and demonstrated its feasibility in a real-world hospital environment. Previous studies have shown that data-driven approaches can support maintenance decision-making and improve equipment reliability; however, most existing work remains focused on retrospective model development or conceptual frameworks rather than operational deployment (Zamzam et al., 2021). By embedding priority assessment into routine maintenance workflows and evaluating downstream operational outcomes, the present study advances prior research toward practical implementation.

Risk-based and priority-oriented maintenance strategies for medical equipment have been widely discussed in the literature, with early frameworks emphasizing equipment criticality, failure risk, and maintenance impact on clinical services (Badnjevic, 2023; Manchadi et al., 2023). More recent studies have incorporated data-driven scoring systems and machine learning models to refine maintenance prioritization (Iadanza et al., 2019; Lin et al., 2024). Nevertheless, most existing approaches adopt unified prioritization frameworks that do not explicitly distinguish PM from CM.

In hospital practice, PM and CM represent fundamentally different decision pathways. Preventive maintenance primarily aims to mitigate risk and plan workloads in advance, whereas corrective maintenance emphasizes rapid response and timely restoration of equipment functionality following failure (Manchadi et al., 2023). The results of the present study support this distinction, as PM and CM devices exhibited different feature distributions, urgency gradients, and clustering patterns. These findings suggest that a single unified prioritization framework may not fully capture the complexity of real-world maintenance decision-making, and that parallel but independent prioritization schemes may be more appropriate for hospital settings.

Machine learning methods have been increasingly applied to maintenance prioritization and failure prediction across healthcare and industrial domains (Lin et al., 2024; Alkhatib et al., 2025). Predictive maintenance approaches, in particular, have been shown to outperform static schedule-based strategies by leveraging historical and operational data to anticipate equipment failures (Lin et al., 2024). Among various algorithms, ensemble learning methods have demonstrated strong performance in handling heterogeneous features and nonlinear relationships commonly observed in maintenance datasets (Moufid et al., 2025).

Consistent with these findings, XGBoost achieved superior and stable performance in both PM and CM classification tasks in the present study. This result supports the suitability of ensemble learning approaches for hospital equipment management, where devices differ substantially in function, usage intensity, and failure patterns. Importantly, the goal of model development in this context is not solely predictive accuracy, but also robustness and consistency across different maintenance scenarios.

Despite the growing body of research on machine learning–based maintenance models, real-world deployment remains relatively limited (Boppana, 2023). Several studies have noted that challenges related to workflow integration, system usability, and organizational adoption often hinder the translation of predictive models into routine clinical engineering practice (Zhu et al., 2025). Reviews of healthcare maintenance strategies further emphasize that technical performance alone is insufficient to ensure real-world impact without effective integration into existing management systems (Li et al., 2023).

In the present study, model-generated priority classifications were embedded directly into routine maintenance workflows, providing actionable guidance for task scheduling and resource allocation. The high adoption rate of model recommendations observed during the implementation period suggests that the system was well aligned with engineering practice and compatible with existing maintenance management processes. These findings highlight the importance of designing decision-support tools that complement, rather than disrupt, established hospital workflows.

Previous studies have suggested that risk-based and predictive maintenance strategies may reduce unplanned downtime, improve maintenance efficiency, and optimize resource utilization in healthcare facilities (Badnjevic, 2023; Alkhatib et al., 2025). However, empirical evidence from real hospital environments remains limited (Iadanza et al., 2019). The results of the present study indicate that priority-based maintenance supported by machine learning was associated with improved maintenance timeliness, fewer unplanned maintenance events, and lower maintenance cost ratios across multiple equipment categories.

Although the magnitude of improvement observed was moderate, the consistency of favorable trends across both preventive and corrective maintenance scenarios suggests meaningful operational benefits. In large tertiary hospitals managing extensive and heterogeneous equipment inventories, even incremental improvements in maintenance efficiency may translate into substantial long-term economic and organizational gains.

An important consideration in applying artificial intelligence to hospital operations is the role of such systems in relation to human expertise. Existing literature emphasizes that intelligent maintenance systems should support, rather than replace, clinical engineers’ professional judgment (Boppana, 2023). By providing structured, transparent, and data-driven priority assessments, the proposed framework enhances situational awareness and reduces variability in maintenance decision-making, while allowing engineers to retain final responsibility for operational decisions.

### 4.1 Limitations and future directions

This study has several limitations. First, it was conducted in a single large tertiary hospital with a relatively mature digital maintenance environment, and its generalizability to hospitals with different equipment compositions, maintenance workflows, and informatics readiness remains to be established. Second, the urgency labels used for supervised learning were derived from unsupervised clustering rather than an external gold standard; therefore, the models should be interpreted as predicting cluster-defined urgency categories rather than independently validated real-world urgency. Third, the real-world deployment evaluation was based on a quasi-experimental campus comparison, so residual confounding cannot be fully excluded and the observed benefits should be interpreted as associative rather than strictly causal. Fourth, the evaluation period was limited and did not allow assessment of longer-term outcomes such as equipment lifespan extension, replacement planning, or sustained cost-effectiveness. Finally, feature weighting was informed partly by expert judgment rather than fully automated optimization, although consistency and sensitivity analyses supported its robustness.

Future research should therefore focus on multi-center external validation, local recalibration in hospitals with different operational contexts, and adaptive updating of prioritization models using continuous feedback. Further work should also explore more automated optimization strategies for weighting and threshold selection, deeper integration with computerized maintenance management systems and hospital information infrastructures, and the incorporation of real-time equipment usage or sensor-based data streams. In addition, future studies should include more comprehensive economic evaluation of the AI-based maintenance framework itself, including implementation cost, computational cost, personnel investment, and long-term cost-effectiveness.

## 5 Conclusion

In summary, this study provides real-world evidence that machine learning–assisted maintenance prioritization can be effectively implemented in hospital environments. By distinguishing preventive and corrective maintenance pathways and embedding model outputs into routine workflows, the proposed framework extends prior methodological research toward practical, outcome-oriented hospital equipment management.

## Data Availability

The raw data supporting the conclusions of this article will be made available by the authors, without undue reservation.
