# Supplementary material for: A data-driven priority assessment and deployment framework for medical equipment maintenance in a tertiary hospital
Source: Front Artif Intell. 2026 Apr 14;9:1791935. doi: 10.3389/frai.2026.1791935 (PMC13121388; doi:10.3389/frai.2026.1791935)

Supplementary Materials

Table S1. Feature Weights Used for Preventive Maintenance Priority Scoring

**Variables**	**Weights Score**
Equipment age	7
Backup or alternative unit	7
Number of missed Planned Preventive Maintenance (PPM)	3
Maintenance requirement	5
Maintenance complexity	3
Downtime	1
Number of failures	7
Function	9

Table S2. Feature Score Used for Preventive Maintenance Priority Scoring

**Variables**	**Group**	**Score**
**Downtime (day)**	0	0
	[1,7]	1
	>7	2
**Function**	Miscellaneous	0
	Monitor	1
	Diagnostic	2
	Therapeutic	3
	Life support	4
**Backup or alternative unit**	No	-1
	Yes	0
**Equipment age**	≤5	0
	（5，10]	1
	>10	2
**Number of failures**	0	0
	[1,5]	1
	> 5	2
**Maintenance complexity**	Self-inspection	0
	Monthly inspection	1
	Daily inspection (	2
**Maintenance requirement**	0	0
	1	1
	2	2
	3	3
**PPM**	0	0
	[1,3]	1
	>3	2

Table S3. Feature Weights Used for Corrective Maintenance Priority Scoring

Variables	Weights Score
Equipment age	7
Response time	1
Maintenance complexity	7
Repair time	3
Number of failures	3
Backup or alternative unit	7
Function	10
Maintenance cost	5

Table S4. Feature Score Used for Corrective Maintenance Priority Scoring

Variables	Group	Score
**Equipment age**	<5	0
	(5,10]	1
	>10	2
**Function**	Miscellaneous	0
	Monitor	1
	Diagnostic	2
	Therapeutic	3
	Life support	4
**Backup or alternative unit**	No	0
	Yes	-1
**Number of failures**	1	0
	[2,5]	1
	>5	2
**Maintenance complexity**	Self-inspection	0
	Monthly inspection	1
	Daily inspection	2
**Response time**	1	0
	[2,7]	1
	>7	2
**Repair time**	1	0
	[2,7]	1
	>7	2
**Maintenance cost(%)**	≤5	0
	（5， 10]	1
	>10	2

Table S5. Distribution of Medical Devices by Functional Category and Device Type

Variables	Group	Number
Miscellaneous (n=4842)	Electronic scales / Infant scales	48
	C-arm / DSA system	378
	Patient warming system	198
	Centrifuge	254
	Incubator	167
	Humidifier	36
	Operating table	178
	Surgical light	177
	Infusion pump	1844
	Physical rehabilitation equipment	209
	Suction device	139
	Microscope	296
	Sterilization equipment	469
	Blood warming device	43
	Medical low-temperature storage equipment	406
Monitor (n=2256)	Patient monitor	1862
	EEG / Sleep monitoring system	50
	Fetal Doppler ultrasound	27
	Electrocardiograph (ECG)	146
	Blood pressure monitoring device	171
Diagnostic (n=357)	CT scanner	19
	Digital radiography (DR)	22
	X-ray system	67
	Ultrasound system	241
	Magnetic resonance imaging (MRI)	8
Therapeutic (n=1909)	CT scannerv	171
	Electrosurgical unit / Ultrasonic scalpel / Gas scalpel	142
	Endoscopic equipment	861
	Physical therapy equipment	598
	Hemodialysis equipment	137
Life support (n=560)	ECMO	7
	Ventilator	371
	Anesthesia machine / Anesthesia system	115
	Infant incubatorv	67
**Total**		**9924**

Table S6. Distribution of Medical Devices by Functional Category Across Hospital Campuses

Campus	Function	Number
**Longwan Campus(n=3503)**	Miscellaneous	1789
	Monitor	718
	Diagnostic	100
	Therapeutic	648
	Life support	248
**Lucheng Campus(n=2805)**	Miscellaneous	1258
	Monitor	740
	Diagnostic	104
	Therapeutic	568
	Life support	135
**Nanpu Campus(n=1584)**	Miscellaneous	732
	Monitor	365
	Diagnostic	92
	Therapeutic	321
	Life support	74
**Oujiangkou Campus(n=2032)**	Miscellaneous	1063
	Monitor	433
	Diagnostic	61
	Therapeutic	372
	Life support	103

Table S7. Cluster validation metrics across candidate K values

Dataset	K	Silhouette Score	Davies-Bouldin Index
**PM**	2	0.61	0.64
	3	0.70	0.48
	4	0.66	0.55
	5	0.59	0.66
**CM**	2	0.58	0.69
	3	0.67	0.50
	4	0.62	0.57
	5	0.55	0.70

Table S8. Inter-expert consistency of feature weighting for preventive and corrective maintenance pathways

Item	Preventive maintenance (PM)	Corrective maintenance (CM)
**No. of candidate variables**	8	8
**No. of experts**	5	5
**Expert qualification**	Senior clinical engineering experts with >10 years of experience in medical equipment maintenance management in tertiary hospitals	Senior clinical engineering experts with >10 years of experience in medical equipment maintenance management in tertiary hospitals
**Scoring range**	1–10	1–10
**Consensus framework**	Delphi-style consultation combined with risk-contribution assessment	Delphi-style consultation combined with risk-contribution assessment
**Kendall's coefficient of concordance (W)**	0.87	0.89
**Interpretation**	Good agreement	Good agreement

Table S9. Sensitivity analysis of urgency classification under weight perturbation in the PM and CM datasets

Dataset	Perturbation scenario	Direction	Perturbation rule	Classification consistency
**PM**	Baseline	–	Original weights	100.0%
**PM**	Low-amplitude perturbation	+	+10%	97.1%
**PM**	Low-amplitude perturbation	−	−10%	97.4%
**PM**	Moderate-amplitude perturbation	+	+20%	92.1%
**PM**	Moderate-amplitude perturbation	−	−20%	92.3%
**PM**	High-amplitude perturbation	+	+30%	86.1%
**PM**	High-amplitude perturbation	−	−30%	86.2%
**CM**	Baseline	–	Original weights	100.0%
**CM**	Low-amplitude perturbation	+	+10%	97.4%
**CM**	Low-amplitude perturbation	−	−10%	97.6%
**CM**	Moderate-amplitude perturbation	+	+20%	92.5%
**CM**	Moderate-amplitude perturbation	−	−20%	92.7%
**CM**	High-amplitude perturbation	+	+30%	86.4%
**CM**	High-amplitude perturbation	−	−30%	86.8%

Table S10. Correlation between weighted total scores and cluster-derived urgency labels

Pathway	Spearman's ρ	P value	Strength of association
**Preventive maintenance (PM)**	0.89	<0.001	Strong positive correlation
**Corrective maintenance (CM)**	0.91	<0.001	Strong positive correlation

Table S11. Class-wise performance metrics of the final XGBoost model in the PM and CM datasets

Dataset	Split	Urgency class	Precision	Recall	Class-wise F1	Macro-F1	Weighted-F1
PM	Training set	Low urgency	0.980	0.989	0.985	0.931	0.952
	Training set	Medium urgency	0.946	0.956	0.951		
	Training set	High urgency	0.876	0.841	0.858		
	Test set	Low urgency	0.965	0.984	0.974	0.884	0.918
	Test set	Medium urgency	0.925	0.910	0.917		
	Test set	High urgency	0.767	0.755	0.761		
CM	Training set	Low urgency	0.976	0.991	0.983	0.874	0.901
	Training set	Medium urgency	0.858	0.867	0.863		
	Training set	High urgency	0.797	0.756	0.776		
	Test set	Low urgency	0.940	0.986	0.962	0.805	0.842
	Test set	Medium urgency	0.815	0.773	0.793		
	Test set	High urgency	0.674	0.646	0.660		

Figure S1. PCA projection of urgency clusters in the CM dataset

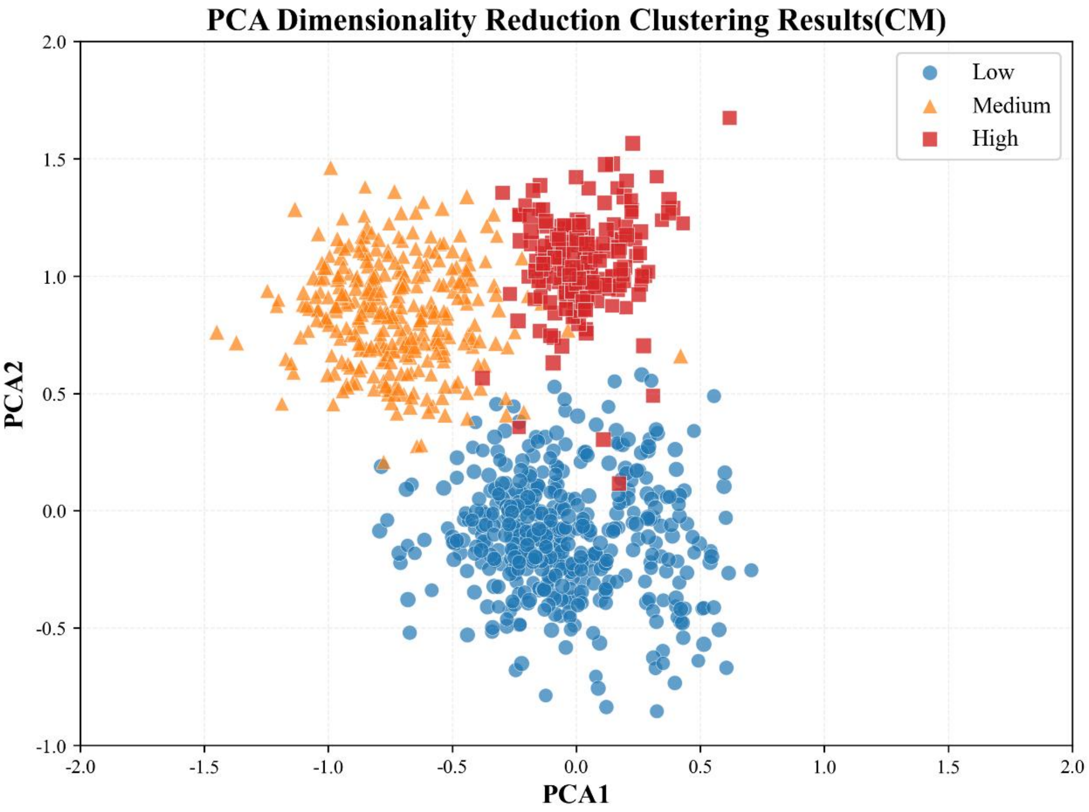

Figure S2. PCA projection of urgency clusters in the PM dataset

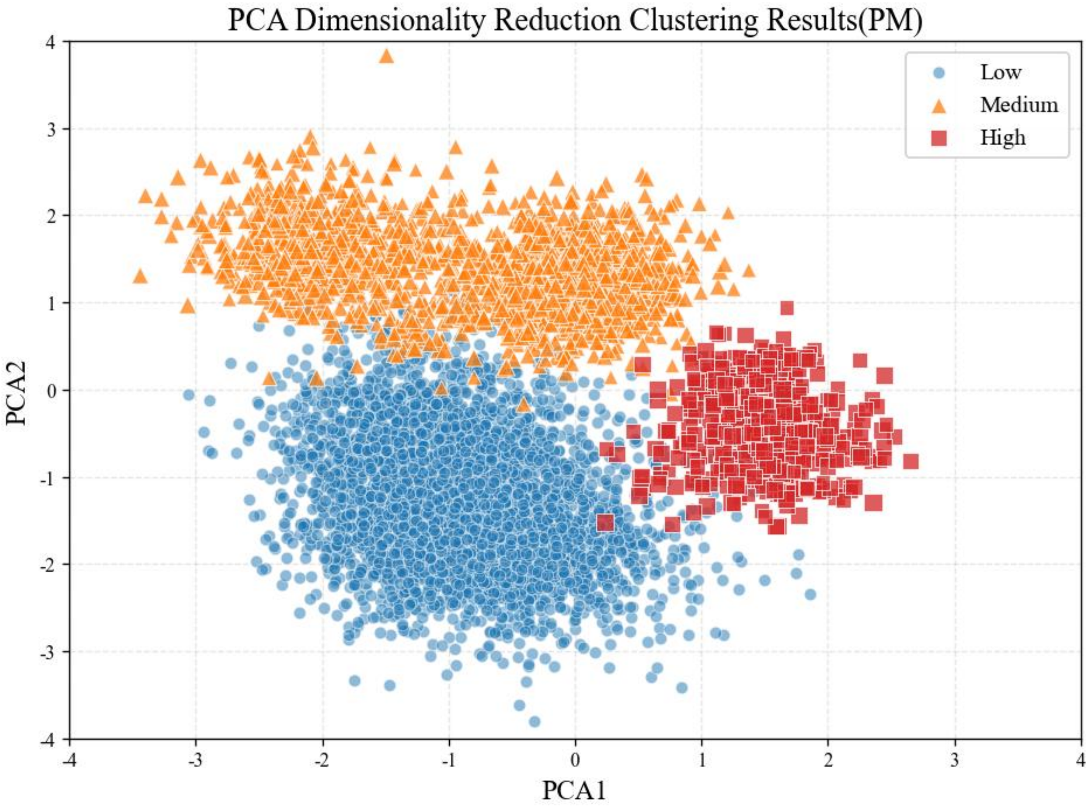

Figure S3. ROC of Corrective Maintenance

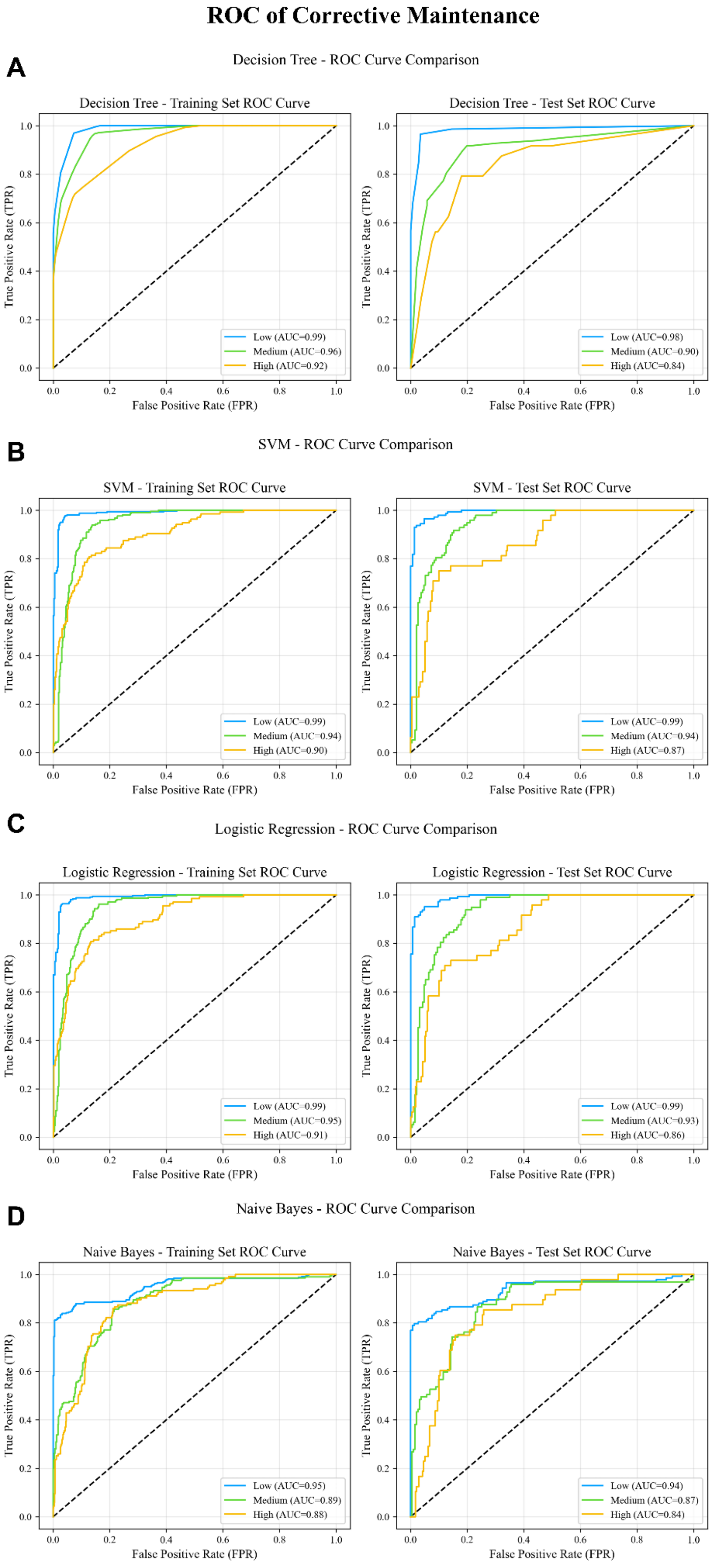

Figure S4. ROC of Preventive Maintenance

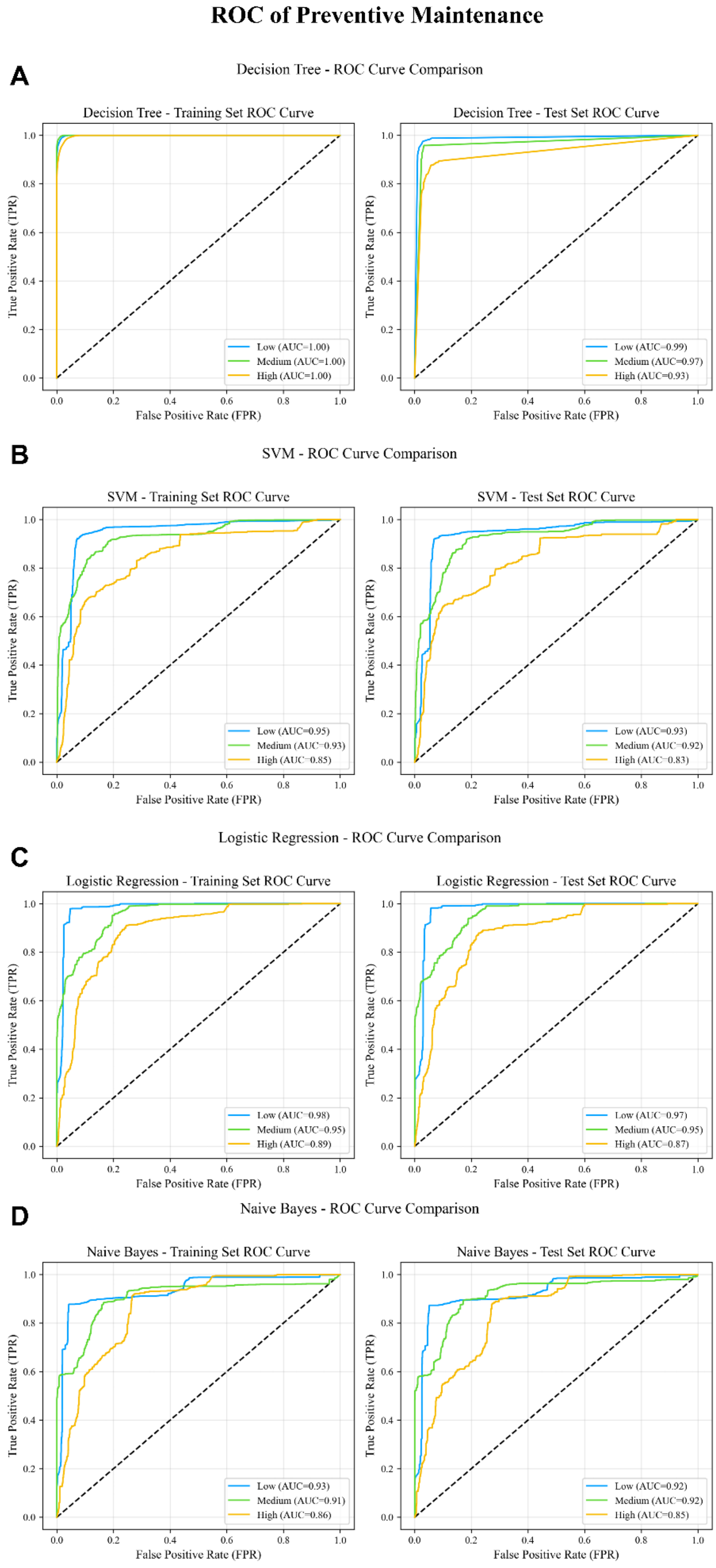

Figure S5. Confusion Matrix Comparison of Corrective Maintenance

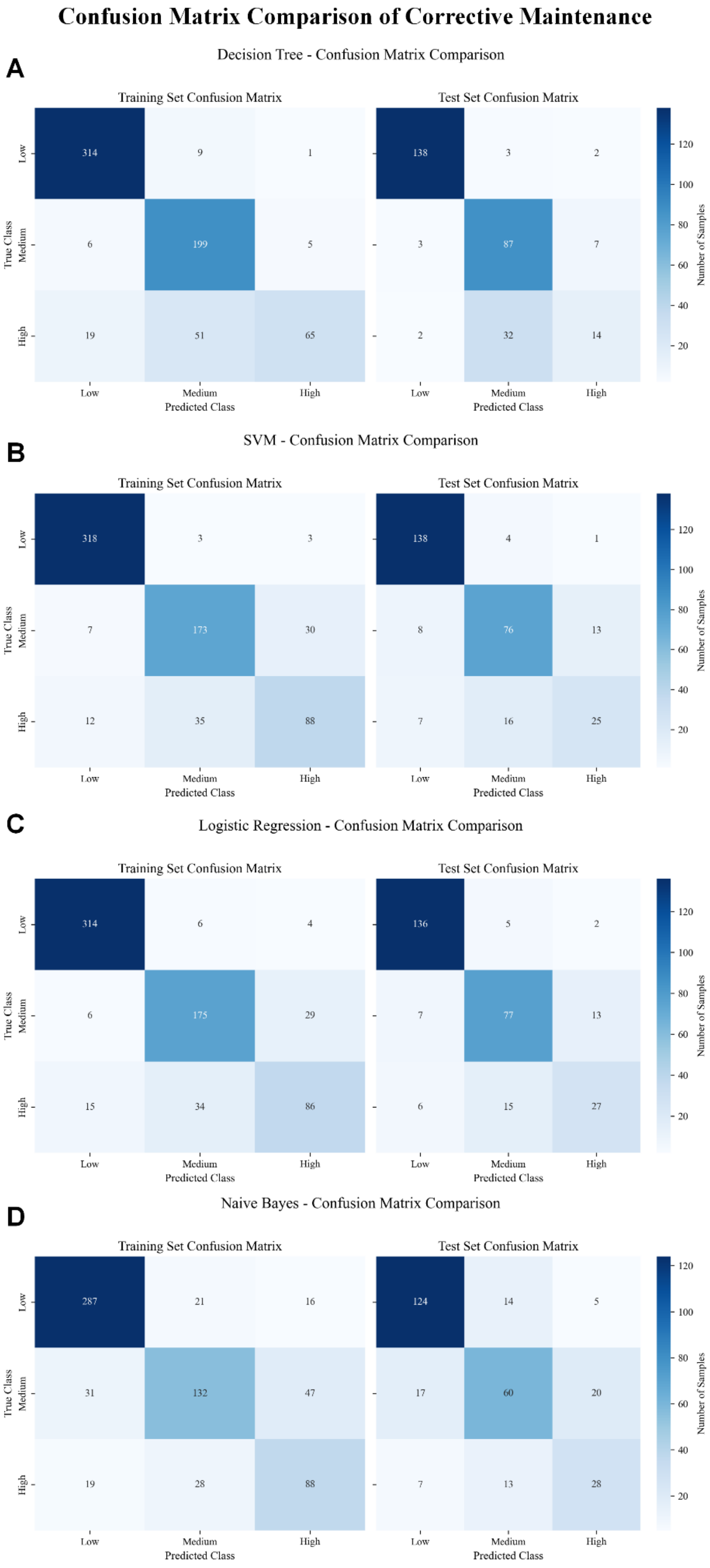

Figure S6. Confusion Matrix Comparison of Corrective Maintenance

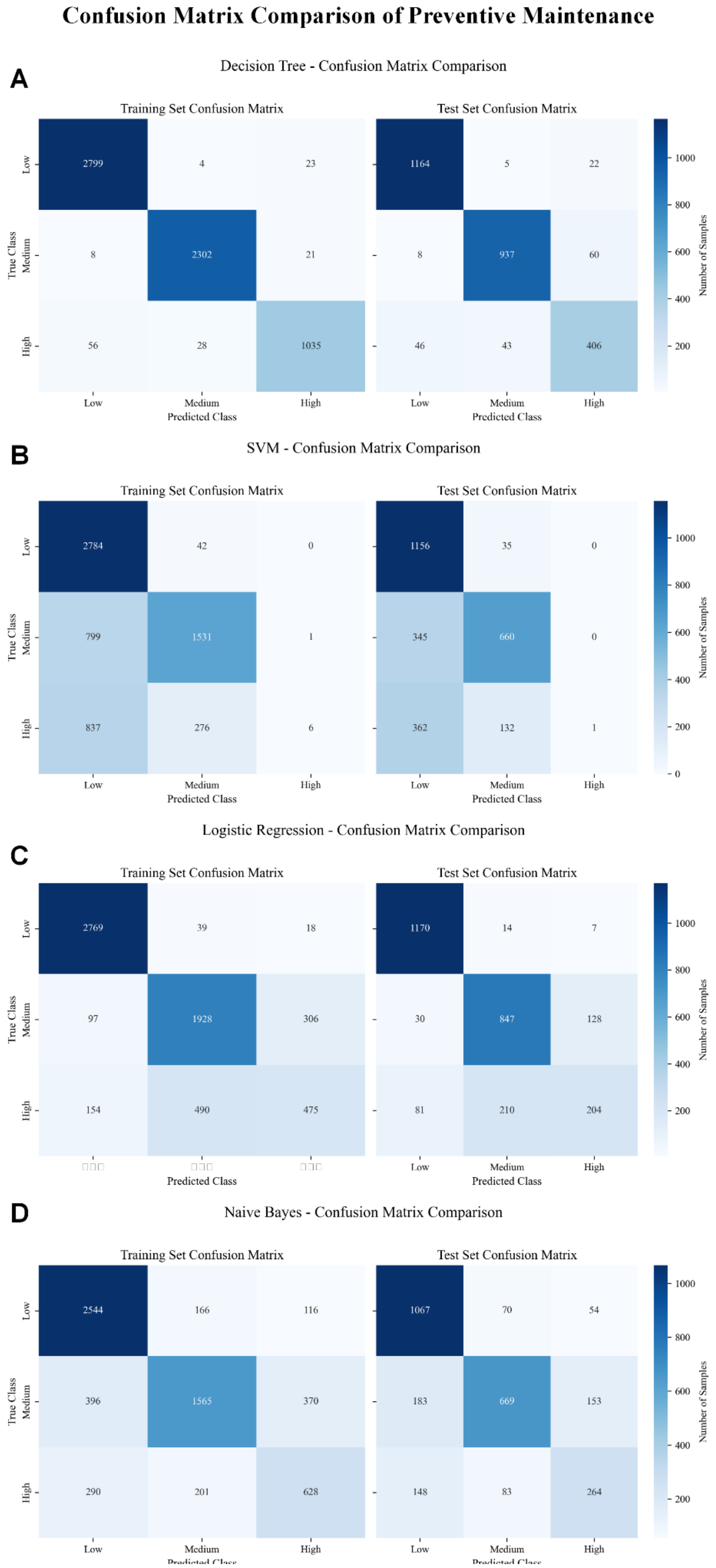

Supplement: Supplementary file 1 [file Data_Sheet_1.pdf]
